# Auxin promotes robust founder cell specification during *Arabidopsis* lateral root initiation

**DOI:** 10.1093/genetics/iyag120

**Published:** 2026-05-11

**Authors:** Cassandra Maranas, Sydney VanGilder, Linda Nguyen, Jennifer Nemhauser

**Affiliations:** Department of Biology, University of Washington, Seattle, WA 98195-1800, United States; Department of Biology, University of Washington, Seattle, WA 98195-1800, United States; Department of Biology, University of Washington, Seattle, WA 98195-1800, United States; Department of Biology, University of Washington, Seattle, WA 98195-1800, United States

**Keywords:** lateral roots, cell differentiation, organogenesis, transition state, cell signaling, auxin signaling, integrase, cell-to-cell variation, cell-to-cell coordination, developmental robustness

## Abstract

Cell-to-cell variation in gene expression can be highly detrimental and, in some contexts, is actively buffered out; however, in other contexts, it is crucial and actively amplified. For example, variation must be minimized to build organs with consistent size and shape, yet the initiation of organogenesis requires a subset of cells to take on a new fate, a process that often relies on small differences between cells. In plants, much of development is controlled by the hormone auxin, which has been hypothesized to coordinate cell responses by inducing degradation of transcriptional repressors. To quantify the level of variation in lateral root initiation and directly test its connection to auxin signaling, we assessed variation in expression of a lateral root founder cell marker *GATA23* when auxin levels or responsiveness was modulated. We found that auxin promoted robustness in lateral root founder cell number in *Arabidopsis thaliana*, as interfering with auxin signaling resulted in more variable numbers of founder cells and, counterintuitively, an increased average number of founder cells per lateral root. These differences were eliminated when using an integrase recorder of *GATA23* expression with an imposed expression threshold instead of a traditional transcriptional reporter. These results led us to posit that auxin acts as both an amplifier and a constrainer of variation during the initiation of a new root. To observe phenotypic effects in a naturally noisier context, we extended this work to analysis of root regeneration, where auxin was also found to affect the robustness of outcomes.

## Introduction

Stochastic cell-to-cell variation in gene expression is well established across organisms and cell types (Elowitz et al. 2002). For many genes, cell-to-cell variation in expression is deleterious to functioning, and cellular mechanisms to buffer it are employed on the gene, transcript, and protein levels (Hong et al. 2016; Urban and Johnston 2018; Fraser et al. 2021; Wu et al. 2022; Munro et al. 2024). However, there is also evidence that expression variability can be advantageous, perhaps even under positive selection (Zhang et al. 2009), and reinforced by multiple regulatory mechanisms (Chalancon et al. 2012). The importance of cell-to-cell variation has been established for many cell differentiation processes, including photoreceptor fate specification in retinal development (Wernet et al. 2006), blastocyst organization (Holmes et al. 2017), and embryonic pancreatic organogenesis (Larsen et al. 2017). These processes often rely on inherent transcriptional noise to trigger cell fate transitions in a subset of cells (Snijder and Pelkmans 2011). In fact, existing cell-to-cell variation can be amplified during organogenesis to form defined boundaries between the identities of differentiating and non-differentiating cells and ensure coordinated development (Urban and Johnston 2018). Studies using single-cell RNA sequencing have identified many genes associated with cell differentiation as being variably expressed (Osorio et al. 2019; Gatlin et al. 2025).

In studies of cell differentiation trajectories, one idea that has been explored is that of a “transition cell state” in which a cell's gene expression profile bears similarities to both the undifferentiated and differentiated cell states and is more heterogeneous than either state (Moris et al. 2016; MacLean et al. 2018). Because of limitations in current experimental techniques for assessing transient cell states, an alternative approach is stochastic modeling of gene expression networks driving differentiation. One such study (Brackston et al. 2018) developed a model of cell differentiation and transition state dynamics based on Waddington's epigenetic landscape (Waddington 1957). In this model, cell states were represented as energy potentials, together forming a probabilistic developmental landscape where cell differentiation was a cell's trajectory through this landscape. Changing the concentration of a signaling molecule distorted the energy landscape of the transition state, and, thus, also distorted cell differentiation trajectories through it. Work like this spotlights the potential relevance of bifurcation theory in describing and modeling the dynamics of complex biological systems. Other work has linked extracellular signaling to heterogeneous transition state dynamics and cell differentiation trajectories (Huang et al. 2007; Chang et al. 2008), but the mechanisms describing how cells variably process these signals to make cell fate decisions are not yet clear.

Despite variability in cell states and differentiation trajectories, development is mostly predictable and robust, resulting in consistently formed tissues and organs. *Arabidopsis* sepal development has been a particularly useful model for studying the relationship between cell-to-cell heterogeneity and developmental robustness. First, in a landmark study, it was shown that when more cells are present (such as in a mature sepal), cell-to-cell variation in growth rates is spatiotemporally averaged, buffering these differences and enabling consistent sepal growth (Hong et al. 2016). In this system, initial cell-to-cell variability is resolved over time and space. Second, rapid and coordinated amplification of cell-to-cell variation is key to sepal initiation, but increasing the speed and sensitivity of the response comes at the expense of cell-to-cell coordination and robustness (Kong et al. 2024). Third, if there is sufficient variation in the earliest stages of sepal development (patterning of sepal primordia sites by the plant hormone auxin), the lack of cell-to-cell coordination is propagated through developmental stages, resulting in inconsistently formed sepals (Trinh et al. 2023; Kong et al. 2024). Taken together, this work shows that proper development is predicated on the management of cell-to-cell variation, first amplifying it to initiate differentiation, and later attenuating it to ensure robust progression through developmental stages.

**Fig. 1.**
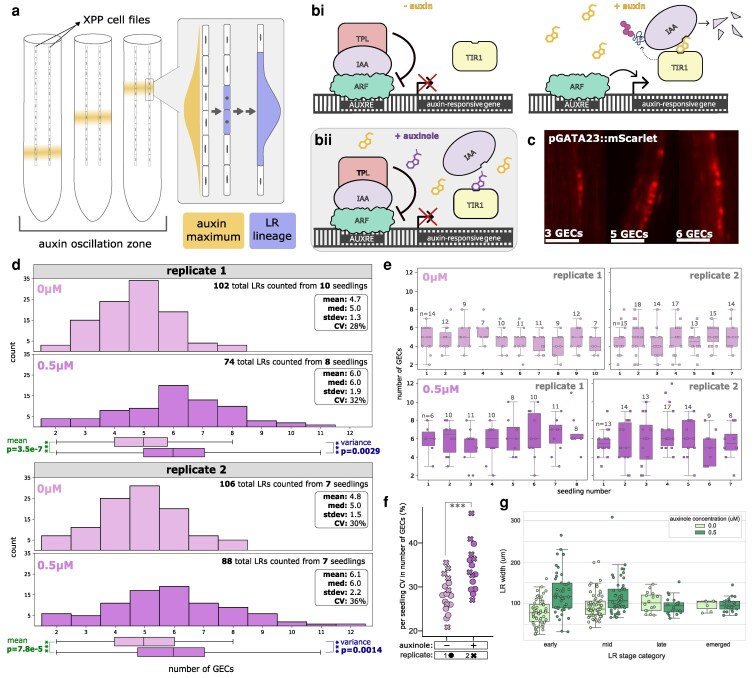
Dampening the auxin signal strength with auxinole treatment increases variability in the early stages of lateral root initiation. a) Specification of LR founder cells. (left) Auxin concentration oscillates along the length of the root defining LR branch sites. (right) A subset of XPP cells which are exposed to high auxin signal undergo a cell fate transition, becoming LR founder cells and dividing to form the LR. bi). Auxin signaling schematics. (left) Without auxin present, TPL represses gene expression through contact with Aux/IAA. (right) When auxin is present, it mediates ubiquitination and degradation of Aux/IAA through contact with TIR1, relieving repression of auxin-responsive genes. bii). With both auxin and auxinole present, auxinole competes for binding with TIR1, reducing IAA degradation rate and slowing relief of repression of auxin-responsive genes. c) Examples of counting GECs using the pGATA23::mScarlet reporter line. The GEC data are used to produce panels d–f). d) Overall distribution in GEC number in the control (light pink) and 0.5 μM auxinole treatment (magenta) for 2 cell counting replicates in WT background. LR and seedling sample size information and summary stats are included for each distribution. Beneath each replicate, a boxplot showing the quartile distribution for each condition is shown. A Student's *t* test was performed for each replicate to compare the means of the GEC distributions for the control and auxinole-treated seedlings with the *P* value shown in green to the left of the boxplots. A Fligner–Killeen test was performed to compare the variance in GEC number between the control and auxinole-treated seedlings with the *P* value shown in navy to the right of the boxplots. The levels of significance for all statistical tests in this work are shown according to the following criteria: ***−*P* < 0.005, **−0.005 < *P* < 0.01, *−0.01 < *P* < 0.05, NS- *P* > 0.05. e) Per seedling distribution in GEC number for control (top) and auxinole (bottom) treatments across 2 replicates. The number of LRs counted for each seedling is indicated above each boxplot. f) Per seedling CV in GEC number for the control and 0.5 μM auxinole treatment. Replicate 1 data is shown with circular data points and replicate 2 with X-shaped points. A student's t test was performed to assess statistical significance with the *P* value being 8.3E-4. g) Overall distribution of LR widths by developmental stage. Stage categories are as follows—early: stages 1–2; mid: stages 3–5; late: stages 6–7, according to Banda et al. (2019).^23^ For early-stage LRs, width was measured based on the extent of GATA23 reporter expression. For the later LR categories, width was measured based on LR protrusion from the main root.

Like sepals, lateral root (LR) development is governed by auxin signaling. Lateral roots are also an excellent model for understanding variation, as their initiation is regulated by one of the best understood plant morphogenetic processes and the roots themselves are completely dispensable in lab-grown plants (Banda et al. 2019). LRs are initiated when a subset of xylem pole pericycle (XPP) cells receive a sufficiently high auxin signal. They then take on an LR founder cell identity and begin to divide (Fig. 1a) (Torres-Martinez et al. 2020). The number of LR founder cells contributing to each LR has been historically described as 2–3 (Van Norman et al. 2013); however, more recent work has revealed that it can vary from as few as 2 to as many as 11 (von Wangenheim et al. 2016).

In the absence of auxin, the expression of auxin-responsive genes is repressed by the TPL/TPR corepressors, which inhibit the activity of AUXIN RESPONSE FACTOR (ARF) activators through association with coreceptors/adaptors from the AUXIN/INDOLE-3-ACETIC ACID (Aux/IAA) family (Fig. 1bi). Auxin signaling facilitates rapid Aux/IAA degradation through formation of a complex between auxin, the Aux/IAA, and auxin receptors like TRANSPORT INHIBITOR RESPONSE 1 (TIR1) (Fig. 1bi). The rate of auxin-induced degradation controls the speed of transcriptional responses (Pierre-Jerome et al. 2014), as well as the rate of LR morphogenesis (Guseman et al. 2015). We hypothesized that, beyond acting as a timer for events within a single cell, auxin might also act to coordinate the response of multiple cells undergoing parallel fate changes during development. To test this hypothesis, we used a variety of chemical and genetic tools to perturb auxin signaling and measure the impact on variation in initiation of a new root in the context of the primary root as well as during regeneration. Our results support a role for auxin as a coordinator of multi-cell behaviors during development.

## Materials and methods

### Plant growth conditions

Arabidopsis seeds were sown in 0.5×Linsmaier and Skoog nutrient medium (LS) (Caisson Laboratories) and 0.8% w/v Phyto agar (PlantMedia/bioWORLD), stratified at 4 °C for 2 d, and grown in constant light at 22 °C.

### Cell counting experiments

Seeds from our cell counting Arabidopsis lines (GATA23 reporter, GATA23 integrase switch34, and GATA23 reporter × IAAslow30) were sown on 0.5 × LS Phyto agar plates as described above, one plate with no auxinole added and one plate with 0.5 μM auxinole (MedChemExpress, solid + solvent). After 8 d of growth in constant light at 22 °C, the plates were scanned using a flatbed 767 scanner (Epson America, Long Beach, CA) to assess root length and emerged LR density for each seedling. Seedlings were mounted on slides using diH2O and imaged with a Leica Biosystems DMI 3000 fluorescent microscope (using the RFP channel for the reporter, CFP and RFP channels for the switch, and the GFP channel for the IAAslow reporter cross line). To enable LR width measurements, for the first GATA23 reporter replicate, an image of every LR on each seedling was taken. For all other replicates, all LRs on each seedling were staged as per Banda et al. (2019), but images were only taken of early-stage LRs (Stage I or II). All LR images were taken at 20 × magnification, with the exception of some LRs with enough *GATA23-*expressing cells (GECs)/*GATA23-*switched cells (GSCs) that reduction to 10 × was needed to fit them all in view. For a given cell counting line, imaging of both the mock and the auxinole-treated seedlings was done on the same day. Two independent replicates were performed on separate days for each line.

### Regeneration assay

Arabidopsis seeds were sown as described above and grown for 12 d in constant light. The first true leaves from 12-d-old seedlings were removed as explants with a double-edged razor blade (Personna). Explants were then cultured on 0.5 × LS plates with 0.8% w/v Phyto agar with concentrations of 0, 1, 2, and 5 μM auxinole. The plates were scanned daily for 13 d of explant culturing using a flatbed 767 scanner and the number of regenerated roots of each explant was also recorded daily. Explants were cultured for an additional week (to day 20) at which point survival, number of regenerated roots, and any callus formation was evaluated and recorded. Callus formation was indicated by a surviving explant without any regenerated roots, with these explants appearing lighter in color and with a brittle texture in comparison to explants with regenerated roots.

### LR/cell counting imaging analysis

All microscope image analysis was performed using the Fiji ImageJ program (version 1.53c). For measuring LR widths the straight line tool was used to span the width of the LR and the measure function was used to measure the distance. For early-stage LRs without protrusion, width was assessed by the extent of bright GATA23 reporter expression. For later-stage LRs, width was measured from the points of protrusion of the LR from the main root. To assess the number of GECs, cells in early-stage LRs were counted as GECs if they were sufficiently brighter in reporter expression than any background level expression in the area. This was determined manually for each LR image by increasing the minimum displayed pixel value in ImageJ until no background expression was visible and then counting the number of *GATA23* expressing cells. Cells were counted as GSCs if they had any mScarlet expression. For Stage I LRs, GEC/GSC number was simply the number of expressing cells in the LR. For Stage II LRs, having undergone the first periclinal division of LR development, GEC/GSC number was assessed by backtracking to the expected number in Stage I, using the well-understood patterns of cell divisions in early LR development. For the IAAslow cross-line, the shape of the nuclei in each imaged LR was also recorded along with GEC number. LRs containing cells with round nuclei were labeled “LR-like,” while LRs containing cells with oblong nuclei were labeled “XPP-like.” LRs containing cells with both appearances were labeled “mixed.” To assess LR density, seedling plate scans were opened in Fiji ImageJ and root lengths were measured using the segmented line tool.

All plots were generated using Python scripts with plotting functions and was run in version 3.9.1 and with the following package dependencies: pandas (version 1.5.3), scipy.stats (version 1.10.0), matplotlib.pyplot (version 3.6.3), matplotlib.colors (version 3.6.3), and numpy (version 1.24.2).

## Results and discussion

As a metric for variation in LR initiation and an early step in auxin signaling, we measured the number of LR founder cells. As *GATA TRANSCRIPTION FACTOR 23* (*GATA23*) expression is one of the earliest markers of LR founder cell identity (Yadav et al. 2010), we counted the number of GECs in early-stage LRs as a proxy for the number of founder cells (Fig. 1c). In our standard growth conditions, the overall distribution of GECs was roughly symmetrical, with a peak around 5, and a range of 2–8, consistent with previous studies (Dubrovsky et al. 2008; Vanstraelen et al. 2012; von Wangenheim et al. 2016). The coefficient of variation (CV), a normalized expression of data spread, makes it possible to compare the extent of variation between different data sets. The CV of GECs in WT plants was 28% (Fig. 1d). We observed some seedling-to-seedling variation in the distribution of GEC number, but the median number of GECs was relatively consistent between seedlings (Fig. 1e). Thus, the observed variability in GEC number is inherent to the LR initiation process, as it is recapitulated in each seedling.

To assess the impact of dampening the auxin signal, we grew seedlings in the presence of low levels of auxinole, a compound that competes with auxin for binding with TIR1 (Fig. 1bii) (Hayashi et al. 2012). To find the ideal concentration for these assays, we quantified emerged lateral root density in a range of doses (Supplementary Fig. 1 in Supplementary File 1). From this result, we selected 0.5 μM auxinole, as it was able to mildly reduce but not block LR development. The distribution of number of GECs in the auxinole-treated LRs was considerably wider than in the control (Fig. 1d), with the maximum increasing to 12 (1.5 × the control) and the median increasing from 5 to 6. There was also a significantly different mean number of GECs in control and auxinole-treated seedlings in each replicate (Fig. 1d). Auxinole treatment also significantly increased the GEC variability by approximately 20%, resulting in a CV of 34%. Computing the CV in GEC number for each seedling and comparing between the control and auxinole treatment (Fig. 1f) shows a significant upwards shift in the CV distribution with auxinole treatment. The increased variability might reflect variable auxinole uptake. However, if this was a major factor, we would expect that other variables that could affect uptake (eg position along the main root) should also affect variability, and we observed no such differences.

Our cell counting approach includes both Stage I and Stage II LRs. To ensure that there are no confounding effects caused by differences between LR stages, we separately plotted the Stage I and II distributions (Supplementary Fig. 3a in Supplementary File 1). We found the biggest difference to be a reduction in low GEC number LRs in Stage II compared to Stage I, which likely reflects that the periclinal cell divisions which initiate stage II are preceded by anticlinal cell divisions during Stage I. Additionally, the relative proportion of Stage I and II LRs was consistent across replicates and treatment conditions (ranging from 51% to 56% Stage I), making confounding effects due to staging differences unlikely. We also wanted to ensure that the slightly reduced number of LRs counted per seedling in the auxinole-treated group did not contribute to the higher observed variation in GEC number. For each of the 2 auxinole treatment datasets, we removed the 2 seedlings with the fewest counted LRs, bringing the average counted LRs/seedling closer to that of the control (Supplementary Table 1 in Supplementary File 1). This modification caused only minimal changes to standard deviation and CV, confirming that reduced LR sample size in auxinole-treated seedlings does not account for the increased variance in GECs.

We were next interested in examining the impact of auxinole treatment in later stages of LR development. To capture LRs across all stages, we measured the width of each LR on each *GATA23* reporter seedling screened. Plotting these LR widths by stage (Fig. 1g), we found that early-stage LRs were the most variable in width, with variation decreasing through each of the later stages. Treatment with auxinole resulted in increased average width and variation therein for early and mid-stage LRs, but in later stages, the distribution grew closer to that of the control. This trend indicates that as LR development progresses, initial cell-to-cell variation is resolved, allowing robust root formation, even when the coordinating auxin signal is dampened by auxinole treatment. This trend could be a function of the increased number of cells in later stage LRs, allowing for spatiotemporal averaging of each cell's growth and cell cycle fluctuations, similar to what is observed in sepal development (Hong et al. 2016).

**Fig. 2.**
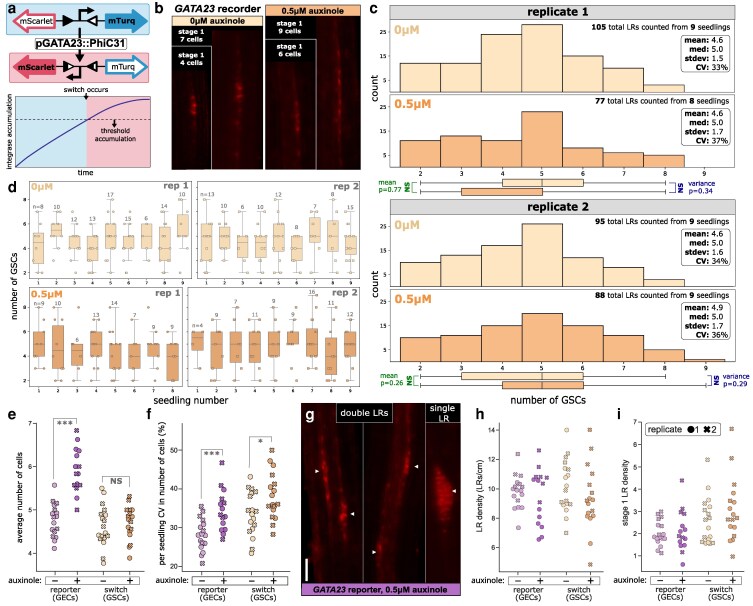
*GATA23* integrase switch shows that dampening the auxin signal during LR initiation results in incomplete repression of the auxin response in neighboring cells and an increased rate of early LR arrests. a) *GATA23* integrase recorder schematic. (left) The recorder starting state is expression of mTurquoise. With sufficient accumulation, the PhiC31 integrase mediates DNA recombination, inverting the promoter direction and switching expression to mScarlet. The switch is permanent, thus mScarlet expression is sustained indefinitely. b) Example images of *GATA23* integrase recorder in early-stage LRs with 0 μM (left) and 0.5 μM (right) of auxinole added. c) Overall distribution in GSC number (as measured by counting the number of mScarlet-expressing cells in the *GATA23* switch line) in the control (light orange) and 0.5 μM auxinole treatment (dark orange) for 2 cell counting replicates. Seedling and LR sample size information and summary stats are included for each distribution. Beneath each replicate, a boxplot showing the quartile distribution for each condition is shown. A Student's t test was performed for each replicate to compare the means of the GEC distributions for the control and auxinole-treated seedlings with the *P* value shown in green to the left of the boxplots. A Fligner–Killeen test was performed to compare the variance in GEC number between the control and auxinole-treated seedlings with the *P* value shown in navy to the right of the boxplots. The levels of significance for all statistical tests in this work are shown according to the following criteria: ***P < 0.005, **0.005 < *P* < 0.01, *0.01 < *P* < 0.05, NS- *P* > 0.05. d) Per seedling distribution in GSC number for control (top) and auxinole (bottom) treatments across 2 replicates. The number of LRs counted for each seedling is indicated above each boxplot. e) Per seedling average number of cells for the *GATA23* reporter (GECs, pink) and recorder (GSCs, orange) in the control and auxinole treatment. Circular data points are from replicate 1 and X data points are from replicate 2. Student's *t* test was performed to assess significance. The *P* values were 5.0E-8 and 0.60 (from left to right). f) Per seedling CV in number of cells for the *GATA23* reporter (GECs, pink) and recorder (GSCs, orange) in the control and auxinole treatment. Student's t test was performed to assess significance. The *P* values were 8.3E-4 and 0.028 (from left to right). g) Example images of “double LRs” (left) seen more frequently in auxinole-treated seedlings and a typical single LR (right) for reference. Arrowheads indicate LRs. Scale bar is 100 μm. h, i) Total (h) and stage I only (i) LR densities for the *GATA23* reporter and recorder, with 0 and 0.5 μM auxinole added.

One model to explain our results is that a weakened auxin response is ineffective in repressing LR fate in cells adjacent to the founder cells, a role that has been previously documented (Cavallari et al. 2021). This is consistent with the observation that auxinole treatment increased the high end of the GEC distribution (Fig. 1d). Following this logic, unrepressed neighboring cells might have lower expression of LR-initiation genes like *GATA23* than cells at the core of the initiating LR. We tested this prediction with a previously characterized integrase-based durable recorder of *GATA23* expression, a tool we call an integrase switch (Guiziou et al. 2023). The integrase switch has 2 components: (i) the target which can switch between expression of one gene to another by inverting the direction of the promoter and (ii) a driver that directs the expression of the PhiC31 serine integrase (Fig. 2a, top). In the integrase switch used here, PhiC31 accumulates in cells when *GATA23* is expressed, and, past a certain accumulation threshold, the target is switched so that cells express mScarlet instead of mTurq. Crucially, unlike a transcriptional reporter, the integrase switch is a binary response: only those cells that surpass a threshold level of *GATA23* expression will switch (GSCs) (Fig. 2a, bottom), and the change in the target and associated reporter expression is permanent and durable in these cells. Previously (Guiziou et al. 2023), we showed that the expression pattern of the *GATA23* recorder is essentially identical to that of a *GATA23* transcriptional reporter in wild-type conditions. Because of the binary nature and imposed expression threshold of the switch, we predicted that the number of GSCs would not be affected by auxinole to the same degree as GECs because the level of *GATA23* expression in ineffectively repressed adjacent cells would likely be below the threshold at which the integrase switch occurs.

We repeated the cell counting process in our *GATA23* switch line using the same approach as with the *GATA23* reporter to evaluate the number of GSCs in early-stage LRs (Fig. 2b). We found there to be a comparable distribution to that of the transcriptional reporter. The distributions of GECs (Fig. 1d) and GSCs (Fig. 2c) had the same median of 5 cells and the same minimum (2) and maximum (8). Seedling-specific plotting of the number of GSCs (Fig. 2d) also reveals similar patterns to that of GECs, with every seedling across both experiments having a median number of cells between 4 and 6. However, unlike the effect seen in GECs (Fig. 1d), application of auxinole did not result in an increase in median or mean number of GSCs (Fig. 2c).

The number of GSCs was not significantly increased with auxinole treatment (Fig. 2c), although there was a modest increase in variability (an overall 6% increase in GSC CV compared to 21% for GECs) (Fig. 2f). On a per seedling basis, each auxinole-treated seedling had a median number of GSCs between 4 and 6 (Fig. 2d), just like the control. As predicted, the effect of auxinole on GSC numbers was weaker compared to GECs. Additionally, as would be expected if auxinole treatment impairs repression of the auxin response in neighboring cells, auxinole treatment led to a higher frequency of “double LRs,” where 2 LRs were formed in very close proximity to one another (Fig. 2g). Loss of function of the peptide RALFL34 is known to interfere with the repression of LR neighboring cells, with the *ralfl34* mutant showing increased divisions of LR-neighboring XPP cells (indicating founder cell identity) and an increased incidence of double LRs (Murphy et al. 2016). Both phenotypes are similar to our results with auxinole treatment (Fig. 2e and g), further supporting a model where auxin signaling levels in central GECs are optimized for lateral inhibition of auxin responsiveness in neighboring cells.

Another difference we noted with the integrase recorder was a higher relative frequency of low GSC numbers. This could be attributed to the same dampened neighbor cell repression if this phenomenon evenly affected LRs regardless of GSC number, but this was not the case. Another possibility is that some proportion of these low GSC LRs are LR terminations. Because many cases of LR arrest occur in early stages, such as through inhibition of LR founder cell division by cytokinin (Li et al. 2006), we posited that the switch line should have a higher proportion of early-stage LRs when compared with the transcriptional reporter. This pattern is consistent with what we observed. While the overall LR density was similar between the switch and reporter lines (Fig. 2h), Stage I density was higher in the switch line (Fig. 2i). Additionally, auxinole treatment increased the relative proportion of Stage I LRs in the recorder seedlings (Supplementary Fig. 2b), but not in the switch seedlings (Supplementary Fig. 2a in Supplementary File 1), suggesting that auxinole treatment increases the likelihood of an LR to terminate early.

In LR development, cytokinin signaling works antagonistically to auxin signaling, and it is the balance between the two that guides root development (Vanstraelen et al. 2012). Mutually antagonistic hormone signaling dynamics are common in plants and are known to affect variability in plant traits, such as in *Arabidopsis* seed germination, where the relative signaling strength of the hormones abscisic acid and gibberellic acid sets the level of variability in germination timing (Abley et al. 2021). Similarly, our results suggest that shifting the balance between auxin signaling and other hormone signaling networks (in this case by reducing auxin signal strength) also affects variability in cell responses.

**Fig. 3.**
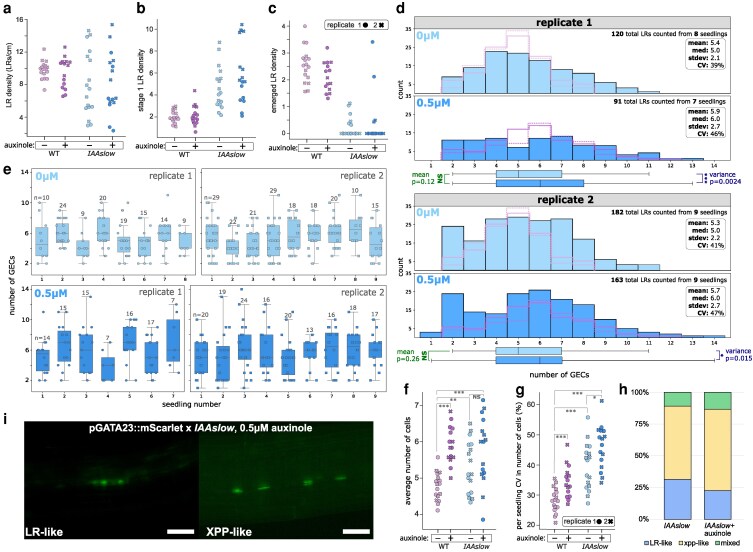
Further dampening of auxin signaling with auxinole and *IAAslow* shows differing effects on GEC number and variation therein, and potentially muddied transition state dynamics. (a–c) Total (a), stage I only (b), and emerged only (c) LR densities in WT and *IAAslow* backgrounds, with 0 and 0.5 μM auxinole added, over 2 replicates. d) Overall distribution in GEC number in *IAAslow* in the control (light blue) and *IAAslow* in 0.5 μM auxinole treatment (dark blue) for 2 cell counting replicates. GECs for the *IAAslow* background were determined by counting GECs in a pGATA23::mScarlet x *IAAslow* cross line. Seedling and LR sample size information and summary stats are included for each distribution. For reference, the 0 and 0.5 μM auxinole distributions of GECs in WT (Fig. 1d) are represented as a dotted (replicate 1) and solid (replicate 2) outlines over the *IAAslow* 0 and 0.5 μM auxinole distributions, respectively. Beneath each replicate, a boxplot showing the quartile distribution for each condition is shown. A Student's t test was performed for each replicate to compare the means of the GEC distributions for the control and auxinole-treated seedlings with the *P* value shown in green to the left of the boxplots. A Fligner-Killeen test was performed to compare the variance in GEC number between the control and auxinole-treated seedlings with the *P* value shown in navy to the right of the boxplots. The levels of significance for both tests are shown according to the following criteria: ***P < 0.005, **− 0.005 < *P* < 0.01, *− 0.01 < *P* < 0.05, NS- *P* > 0.05. e) Per seedling distribution in GEC number in *IAAslow* for control (top) and 0.5 μM auxinole (bottom) treatments across 2 replicates. The number of LRs counted for each seedling is indicated above each boxplot. f) Per seedling average GEC number in WT and *IAAslow* and with 0 and 0.5 μM auxinole treatment. Student's t tests were performed to assess statistical significance and the *P* values from left to right are: 5.0E−8, 0.0092, 1.4E−4, and 0.12 g). Per seedling CV in GEC number in WT and *IAAslow* and with 0 and 0.5 μM auxinole. Student's *t* tests were performed to assess statistical significance and the *P* values from left to right are: 8.3E−4, 8.3E−6, 4.4E−10, and 0.014. h) Proportion of cell appearances in *IAAslow* GECs. i) Example images of LR-like (left) and XPP-like (right) GECs. Scale bar is 100 μm.

As an independent test of the impact of lowered auxin sensitivity on variation, we analyzed a previously characterized line expressing an IAA14 mutant with a lower binding affinity for auxin (*IAAslow*) (Guseman et al. 2015). While the overall LR density in *IAAslow* seedlings was not changed compared to WT, the variation was greatly increased (Fig. 3a). The CV in LR density in *IAAslow* was 42%, compared to 10% in WT. With auxinole treatment, the WT CV in LR density was 15%, which increased to 47% in *IAAslow*. As reported previously, the distribution of LR stages was quite different in *IAAslow* with the majority of LRs in Stage I (Fig. 3b, Supplementary Fig. 2c in Supplementary File 1) and a dramatic reduction in emerged LRs (Fig. 3c, Supplementary Fig. 2c in Supplementary File 1).

Compared to the WT control, we predicted we would find similar increases in mean/median and variability in number of GECs in *IAAslow* as we did with auxinole treatment (Fig. 1d). And indeed, we observed an overall increase in mean and variability in number of GECs in *IAAslow* (mean = 5.3, CV = 40%) compared to the WT control (mean = 4.8, CV = 29%) (Fig. 3c). The range of GEC values also increased in *IAAslow*, with a maximum number of GECs of 11 compared to 8 in WT. The *IAAslow* mutant and auxinole should act independently to reduce auxin sensitivity, so in combination might reveal the upper limits of GEC variation. When *IAAslow* seedlings were exposed to auxinole (*IAAslow* + auxinole), the median for the number of GECs in was 6, an increase from the no auxinole control but relatively unchanged compared to WT + auxinole (Figs. 1d, 3d and e), implying an upper limit to the number of GECs in an initiating LR. The mean showed the same pattern, where the *IAAslow* + auxinole mean of 5.8 GECs was comparable to the WT + auxinole mean of 6.0 GECs (Fig. 3d). The distribution in per seedling average GEC numbers (Fig. 3f) in *IAAslow* + auxinole was not changed compared to *IAAslow* and statistical testing (Fig. 3d) confirms the difference in means is not significant. This contrasts with the upward shift seen in WT + auxinole compared to WT alone (Fig. 3f).

Looking at the variation in the number of GECs during LR initiation, we found that *IAAslow* + auxinole was the most variable, with a CV of 47%. This compares to a CV of 40% for the no auxinole *IAAslow* control and 34% for WT + auxinole. Auxinole significantly increases the variance of GECs in *IAAslow* (Fig. 3d). Plotting CV in GEC number on a per seedling basis for each line and in each auxinole treatment (Fig. 3g), we found that auxinole addition consistently increased the average CV, shifting the distribution upwards in both WT and *IAAslow*. So, while the combinatorial effect on variability in GEC number by *IAAslow* and auxinole was synergistic, the effect on GEC number itself was not. This pattern suggests that there is a functional limit to the number of LR initial cells (a number already close to saturation in both mutant and auxinole treatment), but perhaps not in the potential for variation.

In addition to altered number of GECs, we noticed the majority of the nuclei in GECs in the *IAAslow* background were unusually shaped, often resembling the lens-like shape of nuclei in non-LR XPP cells (Fig. 3i). Fifty-eight percent of the screened LRs were comprised completely of these XPP-like cells and another 10% were comprised of a mix of XPP-like and more typical LR-like cells (Fig. 3h). It could be that these XPP-like GECs represent a transition state within the LR initiation process. Changes to a cell's signaling environment can alter the stability and durability of the transition state (Brackston et al. 2018) and, recently, the capacity of precursor cells to undergo mixed cell fate transitions and the importance of auxin in controlling these transitions has been shown in stomatal development (Tang et al. 2025). In *IAAslow*, a transition state could theoretically arise from a reduction in auxin signal strength and a cell's trajectory could be diverted toward a relatively stable transition state. If this was the case, we would expect that further dampening of the auxin signal with auxinole would increase the likelihood of cells to enter this proposed transition state and we did observe that the auxinole treatment increased the percent of XPP-like LRs to 64% (Fig. 3h).

**Fig. 4.**
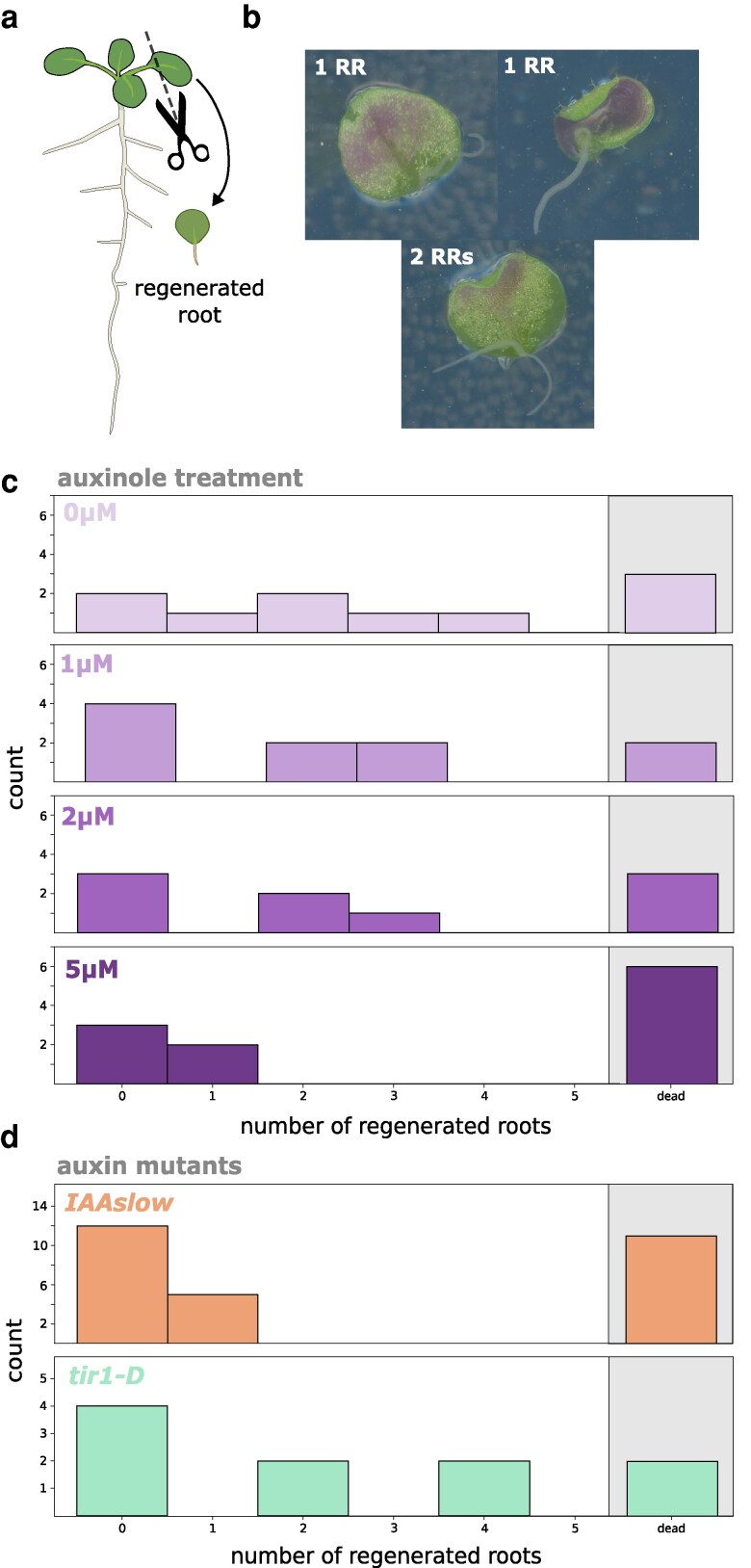
Auxin signaling conditions impact frequency and robustness of root regeneration outcomes. a) Schematic of root regeneraton from *Arabidopsis* leaf explants. The leaf is removed from the seedling and cultured, where it may regenerate a new root/s near the wounding site. b) Example images of 1 RR explants (top), and 2 RR explant (bottom). c, d) Overall distribution in number of RRs for the auxinole treatments c) and the auxin mutants d) taken on day 13.

If auxin is indeed a controller of variation during root initiation, we reasoned that similar trends should be found in regenerated roots (RRs) (Fig. 4a), which involve expression of many of the same developmental genes (Serrano-Ron et al. 2021). In root regeneration from leaf explants, the wound site is flooded with high levels of auxin which are required for RR initiation (Kareem et al. 2025). Root regeneration is well suited to analyzing phenotype variation because, compared to LR development, root regeneration is not robust. Leaf explants of the same age, and even from the same seedling, show natural variation in the number of RRs and the timing of their development (Sugimoto et al. 2010; Xu 2018). Root regeneration offers a naturally noisy root developmental context, making it possible to test whether the effects seen in lateral roots might reflect a more general role for auxin on developmental robustness in root initiation.

We began by testing the effect of different concentrations of auxinole on various metrics of root regeneration, such as timing of RR emergence and number of RRs (Fig. 4b). After removing and culturing the leaf explants, we screened the number of RRs from each explant every day for 13 d. The auxinole treatment delayed the onset of RRs, with the control explants starting to show emerged RRs as early as day 4, while the seedlings treated with 5 μM auxinole began to show emerged RRs on day 6 (Supplementary Fig. 4a in Supplementary File 1). To complement the auxinole treatments, we also analyzed the *IAAslow* mutant and a *tir1*^D170E and M473L^ (hereaffter referred to as *tir1-D*) double mutant, which is hypersensitive to auxin (Yu et al. 2013). In the *IAAslow* background, the earliest emerged RRs were delayed similarly to the auxinole-treated seedlings (Supplementary Fig. 4b in Supplementary File 1). In contrast, the *tir1-D* mutant was not delayed and showed a large jump in the average number of RRs per explant between day 5 and day 7 before leveling off to reach a more modest incline from day 8 to day 13 (Supplementary Fig. 4b in Supplementary File 1). Total number of roots was also correlated to auxin status, with a lower average number of RRs on day 13 for both auxinole treatments and *IAAslow* mutants when compared with control, and a higher average number of roots in *tir1-D* mutants (Supplementary Fig. 4c in Supplementary File 1).

We next plotted the distributions of number of RRs on day 13 for each explant in auxinole treatment (Fig. 4c). In the mock-treated WT, the distribution was widest with explants regenerating anywhere from 0 to 4 roots at comparable frequencies. At the other extreme with 5 μM auxinole, over half the explants did not survive to 13 d, and the ones that did regenerated either 0 or 1 roots. *IAAslow* mutants largely phenocopied the 5 μM auxinole treatment of wild-type plants (Fig. 4d), while the pattern for the *tir1-D* mutants was similar to that seen for intermediate auxinole treatments. Many explants classified as non-regenerating (NR) by day 13 appeared to be forming callus (Ikeuchi et al. 2013) (Supplementary Fig. 4e in Supplementary File 1), an alternative developmental trajectory triggered by auxin treatment (Xu 2018) or wounding (Ikeuchi et al. 2017). We posited that NR explants committed to a callus trajectory would continue to survive without regenerating additional roots, while NR explants not undergoing callus formation would eventually die off. Consistent with the association of callus formation and high auxin levels, increased auxin signaling in *tir1-D* explants led to the highest proportion of surviving NR explants on day 20 (Supplementary Fig. 4d in Supplementary File 1).

## Conclusion

**Fig. 5.**
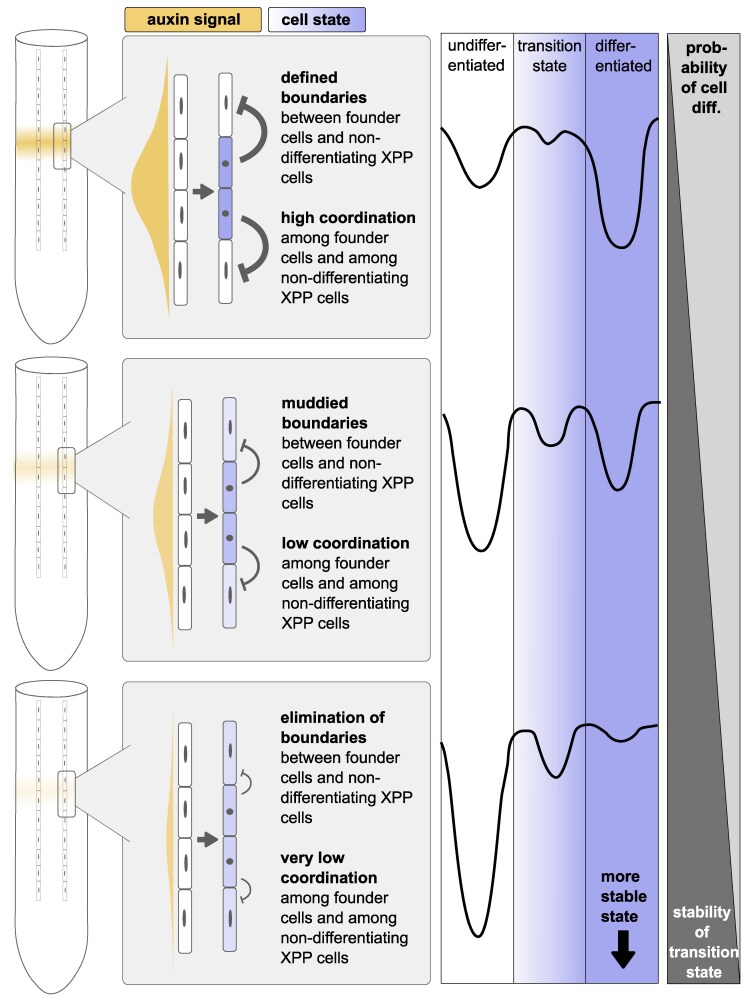
Model of cell differentiation dynamics in different auxin signaling conditions during LR initiation. On the right, graphical representation of the establishment of boundaries between differentiating (purple) and non-differentiating (white) cells with different auxin signal strength (yellow). On the left, representation of energy dynamics underlying undifferentiated, transition, and differentiated states during LR initiation, with deeper valleys representing more stable cell states. Cells are more coordinated when moving through unstable transition states. In a typical auxin signaling context (top) the physical boundaries between XPP cells and LR founder cells is well established through a balance between auxin signal response in founder cells and the repression of this response in neighboring XPP cells. This results in rapid and coordinated progression of founder cells through the transition state to become differentiated. Reduction of the auxin signal (middle) reduces both the strength of the response in founder cells and the repression strength in neighboring XPP cells, resulting in muddied boundaries between differentiating and non-differentiating cells. This reduces the likelihood of differentiation which could be due to stabilization of the undifferentiated and transition cell states and destabilization of the differentiated state. Extreme reduction in the auxin signal (bottom) exacerbates these effects further, resulting in elimination of boundaries and making differentiation even less likely.

Auxin serves to coordinate cell responses both by rapidly turning on LR initiation genes in founder cells (Van Norman et al. 2013), and by repressing the expression of these genes in neighboring XPP cells (Cavallari et al. 2021). Proper balance between these processes is needed to establish defined boundaries between differentiating and non-differentiating cells, both physically and in terms of cell identities. In the WT auxin conditions, robust establishment of these boundaries enables rapid and coordinated progression from an undifferentiated cell state, through the transition state, eventually reaching a differentiated cell state (Fig. 5, top). Our findings suggest that dampening the auxin signal reduces both effects, resulting in a muddying of the cell identities and, therefore, more cells expressing the founder cell marker *GATA23* and increased variability in expression patterns. This dampening, in the case of the *IAAslow* mutant, was associated with increased frequency of *GATA23-*expressing cells, which appeared more like XPP cells than LR cells. We speculated that this effect could be due to stabilized transition state dynamics imbued by the perturbation to auxin signaling, and that these cells represent a transition state in LR development. Overall, in reduced auxin signaling conditions, cells were less likely to differentiate, potentially due to stabilization of the undifferentiated state and the transition state and destabilization of the differentiated cell state (Fig. 5, middle). Further reduction in the auxin signal all but eliminates establishment of boundaries between differentiating and non-differentiating cells, making differentiation much less likely and much more variable if it does occur (Fig. 5, bottom).

Stochastic models of cell differentiation have supported the relationship between signaling conditions and transition state stability and shown that a cell's differentiation trajectory depends on these dynamics (Moris et al. 2016; Brackston et al. 2018; MacLean et al. 2018). Modeling the response of XPP cells to high auxin concentration (Santos Teixeira et al. 2022) has shown that the increased cellular auxin concentration itself is not sufficient to explain the sustained high auxin response required for these XPP cells to become LR founder cells later during development. Instead, the auxin signal prompts these cells to take on a new “primed” cell state, which exhibits a sustained auxin response. This is paired with lateral inhibition mechanisms, which prevent nearby LR formation, which is in line with our hypothesis that LR initiation depends both on amplification and attenuation of the auxin response at different sites.

Cell fate decisions in plant cells are quite plastic, with cells along the same lineage undergoing mixed cell fate transitions in certain circumstances (Tang et al. 2025). Perhaps as a consequence of this plasticity, plant cells also have remarkable regenerative capacity, with differentiated cells under the right conditions becoming totipotent (Xu et al. 2021). Auxin promotes cell plasticity, promoting mixed cell fate decisions and regenerative capacity. Work in *Arabidopsis* sepals has shown that cell-to-cell variability in auxin response is needed to initiate development, yet too much initial variability manifests as loss of phenotype robustness, as the variability is too high to be attenuated through typical strategies.

In plants, there is an established connection between hormone signaling dynamics, cell-to-cell variation, cell differentiation, and developmental outcomes, yet engineering efforts typically focus on individual signaling components to achieve desired phenotypes. Additionally, crops are often intentionally engineered with traits that are optimized for a given growth condition, such as a deeper-penetrating root for drought conditions (Kong et al. 2014), resulting in low variation in these traits, which may not be ideal for growth in increasingly unpredictable environmental conditions due to climate change (Ikeuchi et al. 2017). Approaches targeting cell-to-cell variation could serve as an alternative approach for engineering developmental traits. Variation could be modulated through changes to the growth environment that alter hormone signaling dynamics, and, thus, may also affect cell-to-cell variation and resilience. Alternatively, genetic modifications to components of hormone signaling pathways could be screened for their effect on cell-to-cell variation, with the goal of identifying lines with increased variation which could confer developmental flexibility or resilience to environmental stressors. This engineering philosophy aligns with Ashby's Law of Requisite Variety (Ross Ashby 1958), a foundational principle of cybernetics which states “Only variety destroys variety.” That is, as the complexity of the environment grows, so too must the complexity of the biological response, and engineering cell-to-cell variation could help to achieve this.

## Supplementary Material

iyag120_Supplementary_Data

## Data Availability

Data supporting the findings of this work are available within the paper and its Supplementary Information files. Plasmids and plant materials are available upon request from JLN (jn7@uw.edu; please expect a response within 3 wk). Source data are provided with this paper in Files S2 and S3 and are also available publicly on Dryad (linked here: https://doi.org/10.5061/dryad.g4f4qrg51). Supplemental material is available at GENETICS online.

## References

[iyag120-B1] Abley K et al 2021. An ABA-GA bistable switch can account for natural variation in the variability of Arabidopsis seed germination time. Elife. 10:. 10.7554/elife.59485. PMC8169117 34059197

[iyag120-B2] Banda J et al 2019. Lateral root formation in Arabidopsis: a well-ordered LRexit. Trends Plant Sci. 24:826–839. 10.1016/j.tplants.2019.06.015. 31362861

[iyag120-B3] Brackston RD, Lakatos E, Stumpf MPH. 2018. Transition state characteristics during cell differentiation. PLoS Comput Biol. 14:e1006405. 10.1371/journal.pcbi.1006405. 30235202 PMC6168170

[iyag120-B4] Cavallari N, Artner C, Benkova E. 2021. Auxin-regulated lateral root organogenesis. Cold Spring Harb Perspect Biol. 13:a039941. 10.1101/cshperspect.a039941. 33558367 PMC8247565

[iyag120-B5] Chalancon G et al 2012. Interplay between gene expression noise and regulatory network architecture. Trends Genet. 28:221–232. 10.1016/j.tig.2012.01.006. 22365642 PMC3340541

[iyag120-B6] Chang HH, Hemberg M, Barahona M, Ingber DE, Huang S. 2008. Transcriptome-wide noise controls lineage choice in mammalian progenitor cells. Nature. 453:544–547. 10.1038/nature06965. 18497826 PMC5546414

[iyag120-B7] Dubrovsky JG et al 2008. Auxin acts as a local morphogenetic trigger to specify lateral root founder cells. Proc Natl Acad Sci U S A. 105:8790–8794. 10.1073/pnas.0712307105. 18559858 PMC2438385

[iyag120-B8] Elowitz MB, Levine AJ, Siggia ED, Swain PS. 2002. Stochastic gene expression in a single cell. Science. 297:1183–1186. 10.1126/science.1070919. 12183631

[iyag120-B9] Fraser LCR, Dikdan RJ, Dey S, Singh A, Tyagi S. 2021. Reduction in gene expression noise by targeted increase in accessibility at gene loci. Proc Natl Acad Sci U S A. 118:e2018640118. 10.1073/pnas.2018640118. 34625470 PMC8545487

[iyag120-B10] Gatlin V, Gupta S, Romero S, Chapkin RS, Cai JJ. 2025. Exploring cell-to-cell variability and functional insights through differentially variable gene analysis. NPJ Syst Biol Appl. 11:29. 10.1038/s41540-025-00507-z. 40113778 PMC11926233

[iyag120-B11] Guiziou S, Maranas CJ, Chu JC, Nemhauser JL. 2023. An integrase toolbox to record gene-expression during plant development. Nat Commun. 14:1844. 10.1038/s41467-023-37607-5. 37012288 PMC10070421

[iyag120-B12] Guseman JM et al 2015. Auxin-induced degradation dynamics set the pace for lateral root development. Development. 142:905–909. 10.1242/dev.117234. 25633353 PMC4352979

[iyag120-B13] Hayashi K-I et al 2012. Rational design of an auxin antagonist of the SCFTIR1 auxin receptor complex. ACS Chem Biol. 7:590–598. 10.1021/cb200404c. 22234040

[iyag120-B14] Holmes WR et al 2017. Gene expression noise enhances robust organization of the early mammalian blastocyst. PLoS Comput Biol. 13:e1005320. 10.1371/journal.pcbi.1005320. 28114387 PMC5293272

[iyag120-B15] Hong L et al 2016. Variable cell growth yields reproducible OrganDevelopment through spatiotemporal averaging. Dev Cell. 38:15–32. 10.1016/j.devcel.2016.06.016. 27404356

[iyag120-B16] Huang S, Guo Y-P, May G, Enver T. 2007. Bifurcation dynamics in lineage-commitment in bipotent progenitor cells. Dev Biol. 305:695–713. 10.1016/j.ydbio.2007.02.036. 17412320

[iyag120-B17] Ikeuchi M et al 2017. Wounding triggers callus formation via dynamic hormonal and transcriptional changes. Plant Physiol. 175:1158–1174. 10.1104/pp.17.01035. 28904073 PMC5664475

[iyag120-B18] Ikeuchi M, Sugimoto K, Iwase A. 2013. Plant callus: mechanisms of induction and repression. Plant Cell. 25:3159–3173. 10.1105/tpc.113.116053. 24076977 PMC3809525

[iyag120-B19] Kareem A et al 2025. Water availability positions auxin response maxima to determine plant regeneration fates. Nat Plants. 11:1367–1379. 10.1038/s41477-025-02029-2. 40615607 PMC12283381

[iyag120-B20] Kong S et al 2024. Tradeoff between speed and robustness in primordium initiation mediated by auxin-CUC1 interaction. Nat Commun. 15:5911. 10.1038/s41467-024-50172-9. 39003301 PMC11246466

[iyag120-B21] Kong X, Zhang M, De Smet I, Ding Z. 2014. Designer crops: optimal root system architecture for nutrient acquisition. Trends Biotechnol. 32:597–598. 10.1016/j.tibtech.2014.09.008. 25450041

[iyag120-B22] Larsen HL et al 2017. Stochastic priming and spatial cues orchestrate heterogeneous clonal contribution to mouse pancreas organogenesis. Nat Commun. 8:605. 10.1038/s41467-017-00258-4. 28928395 PMC5605525

[iyag120-B23] Li X, Mo X, Shou H, Wu P. 2006. Cytokinin-mediated cell cycling arrest of pericycle founder cells in lateral root initiation of Arabidopsis. Plant Cell Physiol. 47:1112–1123. 10.1093/pcp/pcj082. 16854941

[iyag120-B24] MacLean AL, Hong T, Nie Q. 2018. Exploring intermediate cell states through the lens of single cells. Curr Opin Syst Biol. 9:32–41. 10.1016/j.coisb.2018.02.009. 30450444 PMC6238957

[iyag120-B25] Moris N, Pina C, Arias AM. 2016. Transition states and cell fate decisions in epigenetic landscapes. Nat Rev Genet. 17:693–703. 10.1038/nrg.2016.98. 27616569

[iyag120-B26] Munro V, Kelly V, Messner CB, Kustatscher G. 2024. Cellular control of protein levels: a systems biology perspective. Proteomics. 24:e2200220. 10.1002/pmic.202200220. 38012370

[iyag120-B27] Murphy E et al 2016. RALFL34 regulates formative cell divisions in Arabidopsis pericycle during lateral root initiation. J Exp Bot. 67:4863–4875. 10.1093/jxb/erw281. 27521602 PMC4983113

[iyag120-B28] Osorio D et al 2019. Single-cell expression variability implies cell function. Cells. 9:14. 10.3390/cells9010014. 31861624 PMC7017299

[iyag120-B29] Pierre-Jerome E, Jang SS, Havens KA, Nemhauser JL, Klavins E. 2014. Recapitulation of the forward nuclear auxin response pathway in yeast. Proc Natl Acad Sci U S A. 111:9407–9412. 10.1073/pnas.1324147111. 24979769 PMC4084466

[iyag120-B30] Ross Ashby W. 1958. Requisite variety and its implications for the control of complex systems. In: Facets of systems science. Cybernetica. p. 83–99.

[iyag120-B31] Santos Teixeira J, van den Berg T, ten Tusscher K. 2022. Complementary roles for auxin and auxin signalling revealed by reverse engineering lateral root stable prebranch site formation. Development. 149:dev200927. 10.1242/dev.200927. 36314783 PMC9793420

[iyag120-B32] Serrano-Ron L et al 2021. Reconstruction of lateral root formation through single-cell RNA sequencing reveals order of tissue initiation. Mol Plant. 14:1362–1378. 10.1016/j.molp.2021.05.028. 34062316 PMC8338891

[iyag120-B33] Snijder B, Pelkmans L. 2011. Origins of regulated cell-to-cell variability. Nat Rev Mol Cell Biol. 12:119–125. 10.1038/nrm3044. 21224886

[iyag120-B34] Sugimoto K, Jiao Y, Meyerowitz EM. 2010. Arabidopsis regeneration from multiple tissues occurs via a root development pathway. Dev Cell. 18:463–471. 10.1016/j.devcel.2010.02.004. 20230752

[iyag120-B35] Tang LP et al 2025. Time-resolved reprogramming of single somatic cells into totipotent states during plant regeneration. Cell. 188:6923–6938.e18. 10.1016/j.cell.2025.08.031. 40961939

[iyag120-B36] Torres-Martínez HH, Hernández-Herrera P, Corkidi G, Dubrovsky JG. 2020. From one cell to many: morphogenetic field of lateral root founder cells in Arabidopsis thaliana is built by gradual recruitment. Proc Natl Acad Sci U S A. 117:20943–20949. 10.1073/pnas.2006387117. 32817465 PMC7456154

[iyag120-B37] Trinh D-C et al 2023. Increased gene expression variability hinders the formation of regional mechanical conflicts leading to reduced organ shape robustness. Proc Natl Acad Sci U S A. 120:e2302441120. 10.1073/pnas.2302441120. 37459526 PMC10372692

[iyag120-B38] Urban EA, Johnston RJ Jr. 2018. Buffering and amplifying transcriptional noise during cell fate specification. Front Genet. 9:591. 10.3389/fgene.2018.00591. 30555516 PMC6282114

[iyag120-B39] Van Norman JM, Xuan W, Beeckman T, Benfey PN. 2013. To branch or not to branch: the role of pre-patterning in lateral root formation. Development. 140:4301–4310. 10.1242/dev.090548. 24130327 PMC4007709

[iyag120-B40] Vanstraelen M, Benková E. 2012. Hormonal interactions in the regulation of plant development. Annu Rev Cell Dev Biol. 28:463–487. 10.1146/annurev-cellbio-101011-155741. 22856461

[iyag120-B41] von Wangenheim D et al 2016. Rules and self-organizing properties of post-embryonic plant organ cell division patterns. Curr Biol. 26:439–449. 10.1016/j.cub.2015.12.047. 26832441

[iyag120-B42] Waddington CH . 1957. The strategy of the genes: a discussion of some aspects of theoretical biology. Allen & Unwin.

[iyag120-B43] Wernet MF et al 2006. Stochastic spineless expression creates the retinal mosaic for colour vision. Nature. 440:174–180. 10.1038/nature04615. 16525464 PMC3826883

[iyag120-B44] Wu H-W et al 2022. Noise reduction by upstream open reading frames. Nat Plants. 8:474–480. 10.1038/s41477-022-01136-8. 35501454 PMC9122824

[iyag120-B45] Xu L . 2018. De novo root regeneration from leaf explants: wounding, auxin, and cell fate transition. Curr Opin Plant Biol. 41:39–45. 10.1016/j.pbi.2017.08.004. 28865805

[iyag120-B46] Xu M, Du Q, Tian C, Wang Y, Jiao Y. 2021. Stochastic gene expression drives mesophyll protoplast regeneration. Sci Adv. 7:eabg8466. 10.1126/sciadv.abg8466. 34380624 PMC8357238

[iyag120-B47] Yadav SR, Bishopp A, Helariutta Y. 2010. Plant development: early events in lateral root initiation. Curr Biol. 20:R843–R845. 10.1016/j.cub.2010.09.010. 20937469

[iyag120-B48] Yu H et al 2013. Mutations in the TIR1 auxin receptor that increase affinity for auxin/indole-3-acetic acid proteins result in auxin hypersensitivity. Plant Physiol. 162:295–303. 10.1104/pp.113.215582. 23539280 PMC3641209

[iyag120-B49] Zhang Z, Qian W, Zhang J. 2009. Positive selection for elevated gene expression noise in yeast. Mol Syst Biol. 5:299. 10.1038/msb.2009.58. 19690568 PMC2736655

