# Supplementary material for: Auxin promotes robust founder cell specification during Arabidopsis lateral root initiation
Source: Genetics. 2026 May 11;233(3):iyag120. doi: 10.1093/genetics/iyag120 (PMC13334086; doi:10.1093/genetics/iyag120)
Supplement: iyag120_Supplementary_Data [file iyag120_supplementary_data.zip › File_S1_GENETICS-2026-309254.docx]

**Supplementary Material**

**Table S1:** Summary stats for GEC/GSC distributions, including all auxinole-treated seedlings and omitting those with the fewest LRs, formatted in each cell as “replicate 1, replicate 2”.

|  | **WT GECs** | | **WT GSCs** | | **IAA slow GECs** | |
| --- | --- | --- | --- | --- | --- | --- |
|  | Inc. all seedlings | Omitting low LR seedlings | Inc. all seedlings | Omitting low LR seedlings | Inc. all seedlings | Omitting low LR seedlings |
| mean | 6.0, 6.1 | 6.0, 6.0 | 4.6, 4.9 | 4.6, 4.9 | 5.9, 5.7 | 6.0, 5.6 |
| median | 6.0, 6.0 | 6.0, 6.0 | 5.0, 5.0 | 5.0, 5.0 | 6.0, 6.0 | 6.0, 6.0 |
| std dev | 1.9, 2.2 | 2.0, 2.1 | 1.7, 1.7 | 1.7, 1.8 | 2.7, 2.7 | 2.7, 2.7 |
| CV (%) | 32, 36 | 33, 36 | 37, 36 | 37, 36 | 46, 47 | 44, 48 |
| num LRs | 74, 88 | 60, 71 | 77, 88 | 64, 77 | 91, 163 | 77, 136 |
| num seedlings | 8, 7 | 6, 5 | 8, 9 | 6, 7 | 7, 9 | 5, 7 |
| avg LRs /seedling  (auxinole) | 9.3, 12.6 | 10, 14.2 | 9.6, 9.8 | 10.7, 11 | 13, 18.1 | 15.4, 19.4 |
| avg LRs/ seedling (control) | 10.2, 15 | - | 11.1,10.6 | - | 15, 18.7 | - |


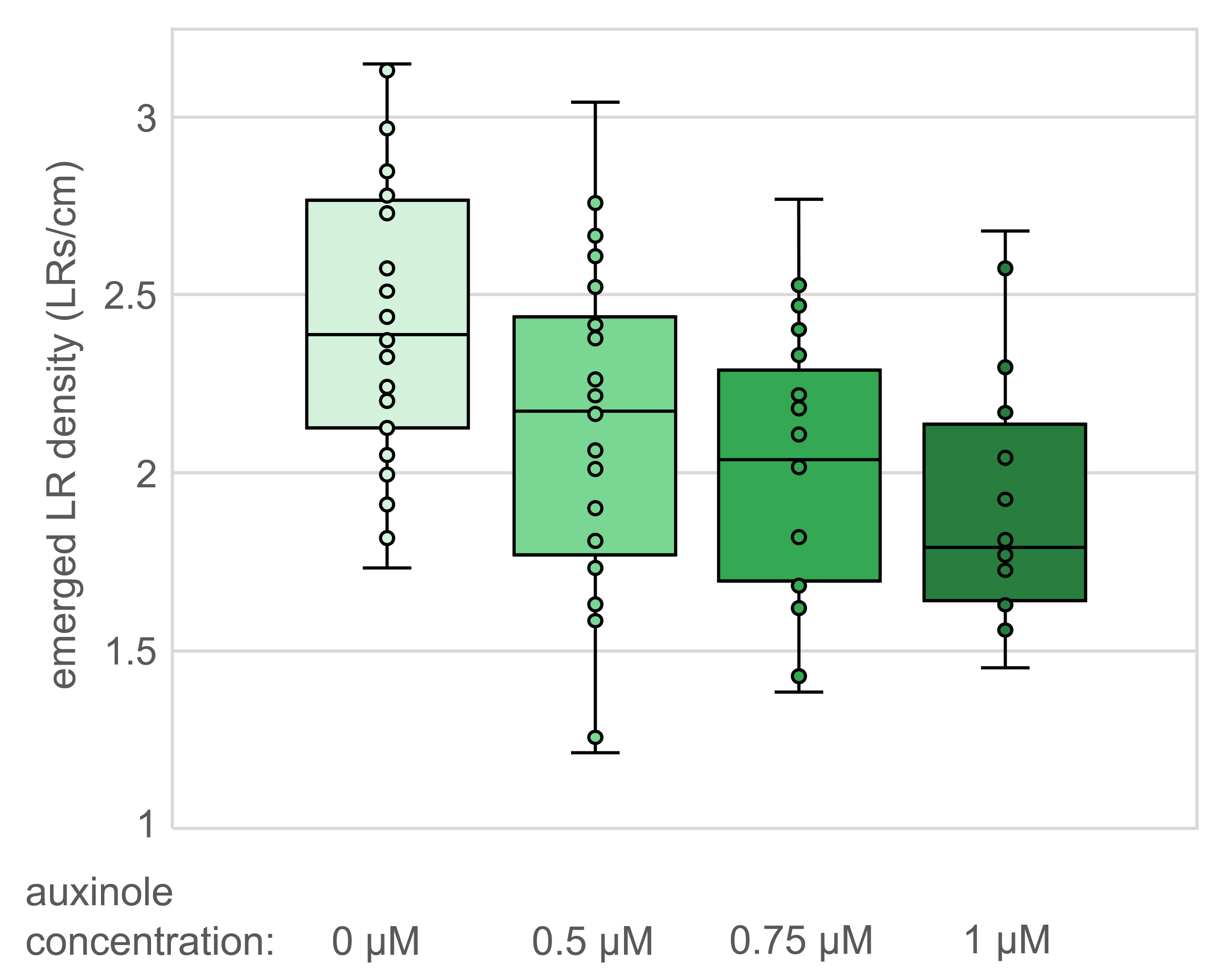


**Figure S1: Dose response for emerged LR density in different auxinole concentrations.** Seedlings were screened for the number of emerged LRs per cm of main root in 0, 0.5, 0.75, and 1 μM auxinole concentrations. Each point represents the LR density of one seedling in the given auxinole condition.

**
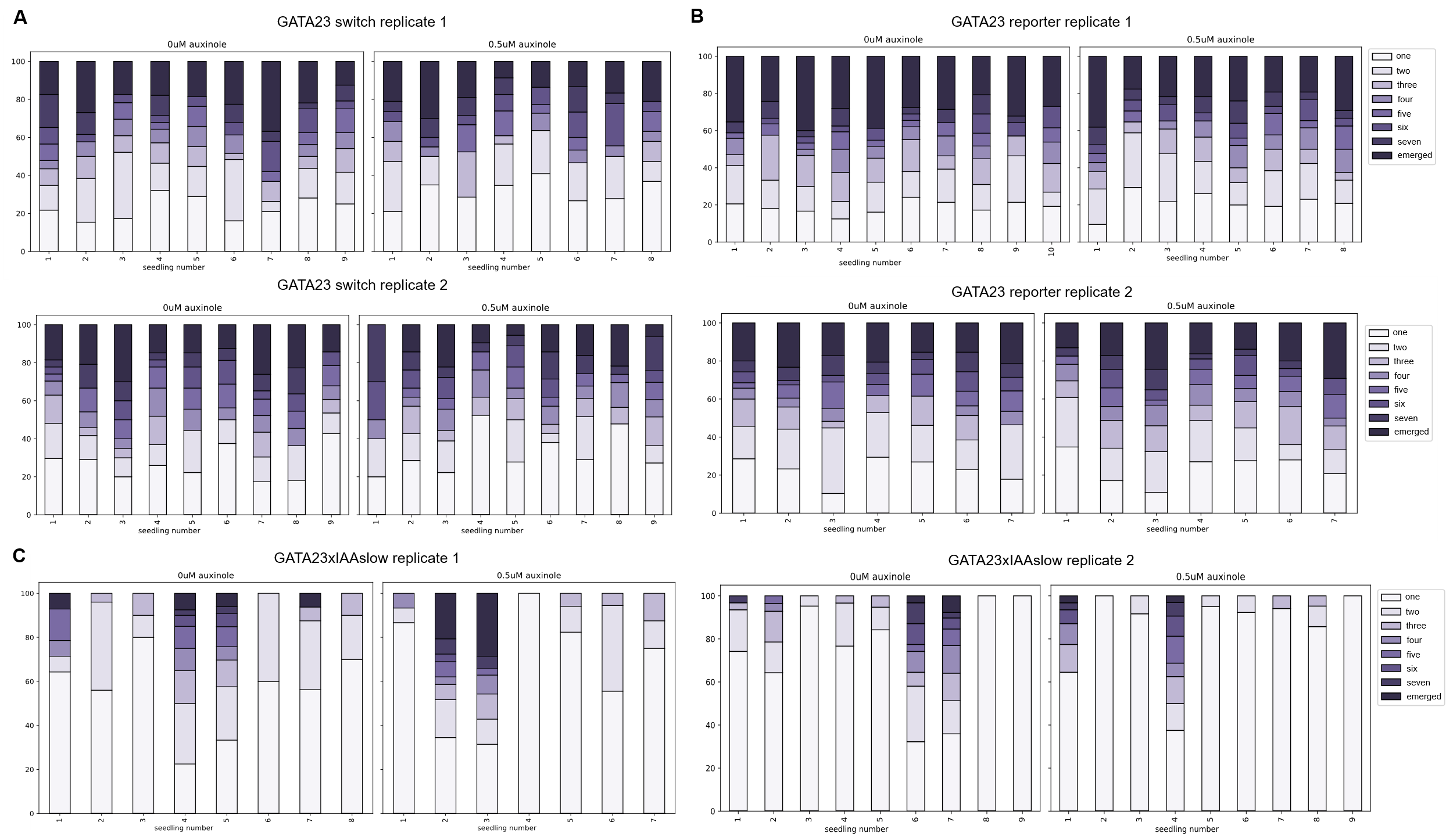
**

**Figure S2: LR staging distributions for *GATA23* switch, *GATA23* reporter, and *GATA23xIAAslow*, auxinole treatment and control.** LR staging distributions for each screened *GATA23* switch (**A**), *GATA23* reporter (**B**), and *GATA23xIAAslow* (**C**) seedling across 2 replicates and treated with 0 and 0.5 μM auxinole. Later LR stages are indicated with darker shades of purple as indicated in the legend.

**
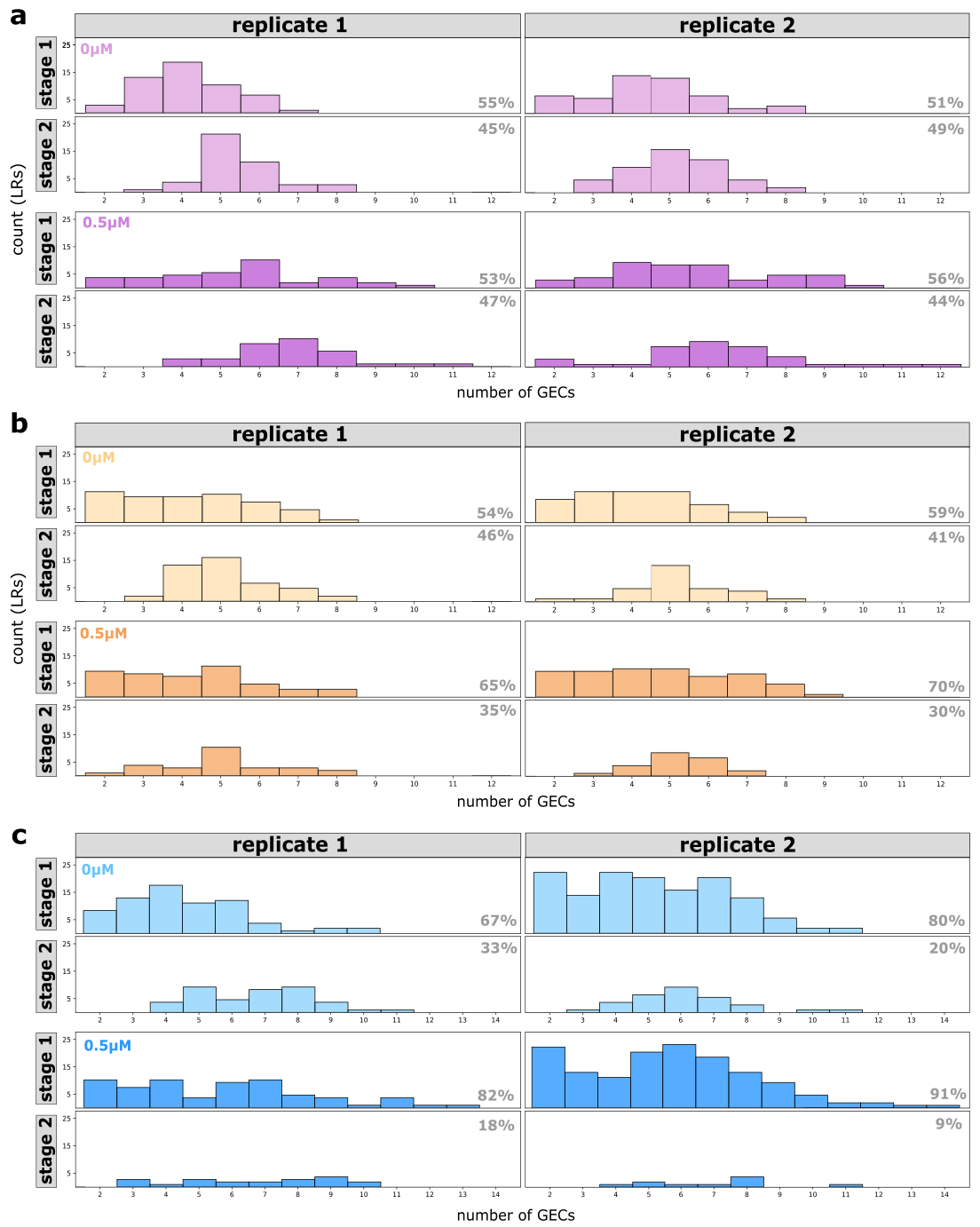
**

**Figure S3**: **Cell number distributions among Stage I and Stage II LRs.** Histograms showing, among Stage 1 and among Stage II LRs, the distribution of (**A**) WT GEC number, (**B**) WT GSC number using the *GATA23* recorder, and (**C**) *IAAslow* GECs. Both replicates (left vs. right) and treatment conditions (0 or 0.5 μM auxinole) are shown for each. At the right of each plot, the percentage of counted LRs of each stage (I or II) is shown.

**
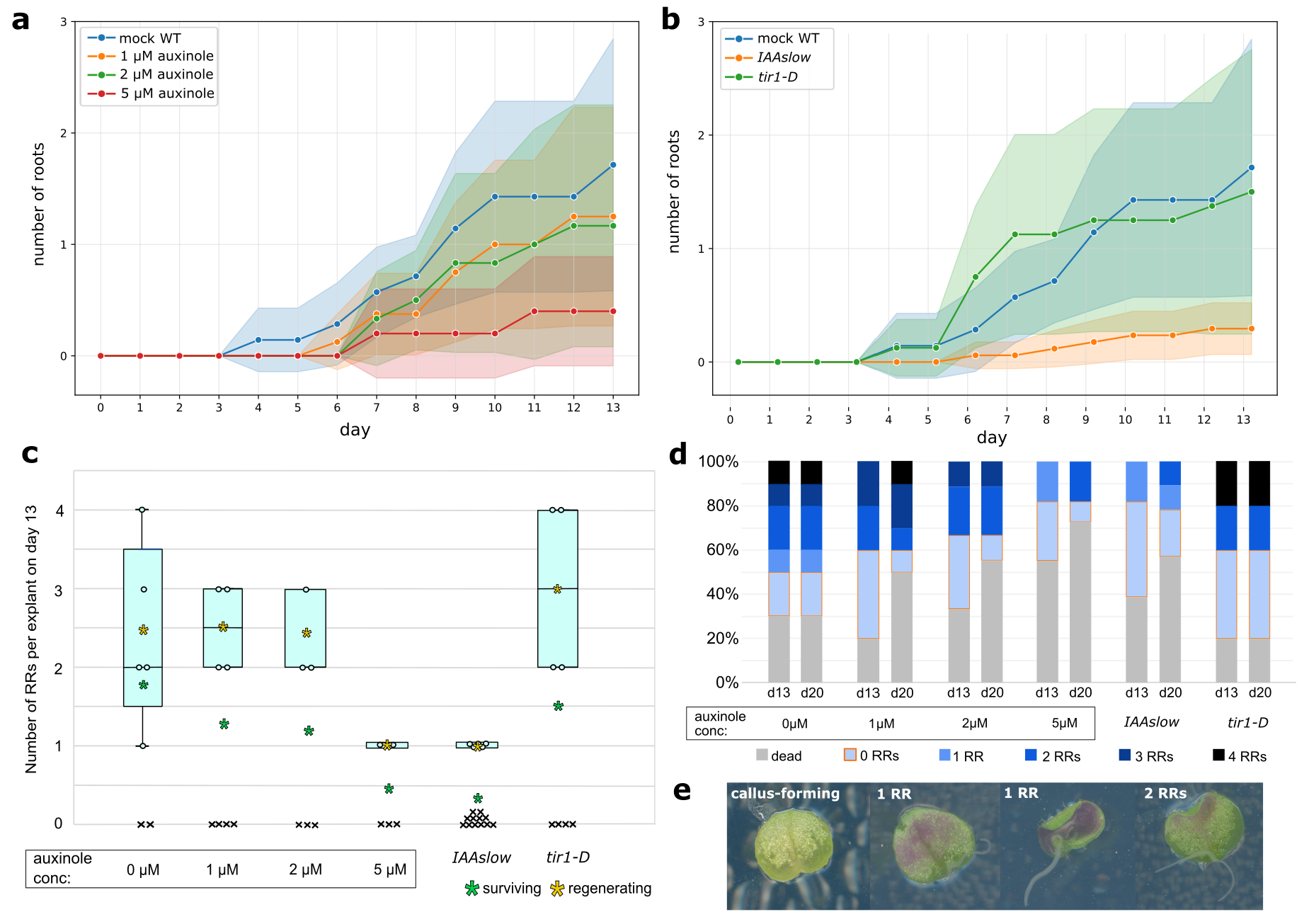
**

**Figure S4: Root regeneration outcomes continued**. **A, B.** Regeneration timelines for (**A**) auxinole-treated and (**B**) auxin mutant explants. Day 0 is the day of explant excision. Shaded areas represent standard error. **C.** Distribution for the number of RRs on day 13 for each auxinole treatment and auxin mutant line. Each point is the number of RRs for a single explant. The green asterisks represent the average number of RRs among all surviving explants in each group, and the yellow asterisks represent the average when including only explants with at least one RR at day 13. The x data points represent surviving explants which regenerated no roots. **D.** Changes in regeneration outcomes from day 13 (d13) to day 20 (d20) for each auxinole treatment and auxin mutant. **E.** Example images of a callus-forming explant (left), 1 RR explant (middle), and 2 RRs explant (right).
